# A rapid RIG-I signaling relay mediates efficient antiviral response

**DOI:** 10.1016/j.molcel.2022.11.018

**Published:** 2022-12-06

**Authors:** Daniel T. Thoresen, Drew Galls, Benjamin Götte, Wenshuai Wang, Anna M. Pyle

**Affiliations:** 1 Department of Molecular, Cellular and Developmental Biology, Yale University, New Haven, CT, USA; 2 Department of Chemistry, Yale University, New Haven, CT, USA; 3 Howard Hughes Medical Institute, Chevy Chase, MD, USA; 4 Lead contact

## Abstract

RIG-I is essential for host defense against viral pathogens, as it triggers the release of type I interferons upon encounter with viral RNA molecules. In this study, we show that RIG-I is rapidly and efficiently activated by small quantities of incoming viral RNA and that it relies exclusively on the constitutively expressed resident pool of RIG-I receptors for a strong antiviral response. Live-cell imaging of RIG-I following stimulation with viral or synthetic dsRNA reveals that RIG-I signaling occurs without mass aggregation at the mitochondrial membrane. By contrast, interferon-induced RIG-I protein becomes embedded in cytosolic aggregates that are functionally unrelated to signaling. These findings suggest that endogenous RIG-I efficiently recognizes viral RNA and rapidly relays an antiviral signal to MAVS via a transient signaling complex and that cellular aggregates of RIG-I have a function that is distinct from signaling.

## In brief

Thoresen et al. show that the resident pool of cytoplasmic RIG-I readily detects small quantities of viral RNA, inducing a rapid antiviral response that does not involve massive aggregation at the mitochondria membrane. In contrast, when the RIG-I gene is induced by interferon, RIG-I forms signaling-unrelated cytosolic aggregates.

## Graphical abstract

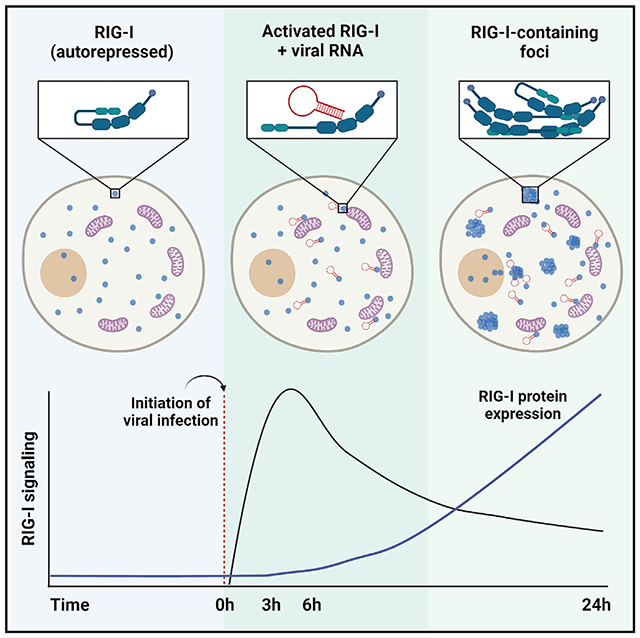

## INTRODUCTION

RIG-I is a cytosolic innate immune receptor that plays a central role in our ability to respond rapidly to infections by RNA viruses.^1–4^ This large, multi-domain protein differentiates viral from host RNA by specifically recognizing blunt-ended, double-stranded RNAs containing 5’ di- or tri-phosphates, which are common features of viral RNA genomes and viral replication intermediates.^5–9^ The binding of RNA viral ligands causes RIG-I to extend its N-terminal caspase activation and recruitment domains (CARDs)^10^ into solution, where they are then free to interact with homologous CARDs on the mitochondrial antiviral signaling protein (MAVS).^11,12^ The interaction between RIG-I and MAVS is thought to initiate the assembly of a MAVS oligomeric complex^13^ that stimulates translocation of transcription factors IRF3 and NF-κB^14–16^ into the nucleus, resulting in the production of type I interferons and interferon-stimulated genes (ISGs). Two critical events are needed to initiate the process of RIG-I signaling upon infection: (1) binding of viral RNA to RIG-I, with concomitant release of the CARDs, and (2) formation of CARD-CARD interaction between RIG-I and MAVS.

Given the immediate danger posed by viral threats, RIG-I signaling must have the following characteristics: First, it must be highly efficient in sensing the first viral RNAs that have infected the cell. Second, it must be extremely rapid to initiate the cascade of signaling reactions and transcriptional events required for alerting the immune system as quickly as possible. Consistent with the need for speed and efficiency, studies of RIG-I signaling *in cellulo* have previously shown interferon response to viral infection within 2h,^17,18^ and active RIG-I signaling *in vivo* has been observed within 2 h after injection of RIG-I-specific RNAs.^8^ Unfortunately, previous studies on subcellular behavior of RIG-I and its responses to RNA activation, including available studies on RIG-I localization, have been conducted at very late time points, ranging from 10–40 h after introduction of viral RNA into cells.^19–21^ These late snapshots of RIG-I signaling, together with *ex vivo* biochemical and biophysical studies performed with transfected or recombinant protein, have led to a “massive oligomer” model for RIG-I-MAVS signal transmission.^22^ According to this model, RNA-stimulated populations of RIG-I migrate *en masse* to the mitochondrial network or to the mitochondria-associated membrane (MAM) of the ER,^20,23^ where RIG-I becomes enmeshed within a large prion-like MAVS aggregate that is required for signal transduction.^13^ This subcellular structure is thought to be so large that it must ultimately be cleared by autophagy.^24^ However, unlike other innate immune signaling complexes, such as the inflammasome^25^ and myddosome,^26^ direct visualization of functional RIG-I signaling complexes has been elusive in cells. Indeed, RIG-I signaling complexes have never been characterized or studied *in cellulo* within the short time frame that is suggested by *in vivo* studies of RIG-I function.

Not only does RIG-I trigger the initial interferon response upon infection by RNA viruses, it is itself an ISG. Given this behavior, it has been suggested that interferon-induced RIG-I expression can boost the antiviral response by supplying additional receptors that bind viral RNA and amplify RIG-I signaling, creating a late-stage second wave of interferon induction and a positive feedback loop. Indeed, this “priming” of RIG-I signaling by interferon has been proposed to play a central role in antiviral signaling.^27,28^ However, naive and unprimed cells must be able to respond to viral infection and trigger a robust immune response or an initial infection would escape detection.^17,29^ Therefore, the role of downstream, interferon-induced RIG-I expression remains unclear.

To address these gaps in our understanding and reveal the dynamic behavior of functional RIG-I molecules as they actively engage in signaling, we measured endogenous RIG-I signaling kinetics and efficiency in response to both synthetic and viral pathogen-associated molecular patterns (PAMPs). We then linked these measurements with direct observation of endogenous RIG-I via fluorescence confocal microscopy in order to observe the receptor distribution during active signaling. Here, we demonstrate that not only is RIG-I signaling both rapid and efficient *in cellulo* but that the active RIG-I signal is transmitted to MAVS without visible RIG-I accumulation on the mitochondrial network. We observe two separate phases of RIG-I behavior upon activation by viral RNA ligands. The “early phase” involves a burst of RIG-I signaling and IFN induction that is initiated within an hour and which is not accompanied by increases in RIG-I protein expression. The “late phase” of RIG-I induction involves dramatic increases in RIG-I expression but coincides with a decline in signaling and the production of type I interferon. Rather than accumulating at the mitochondria at long time points after RNA induction, RIG-I forms subcellular bodies at other positions within the cytosol, none of which are involved in RNA-stimulated RIG-I signaling. Importantly, we show that blocking interferon-induced RIG-I protein expression does not disrupt robust RNA-induced RIG-I signaling, thereby demonstrating that the resident cytosolic pool of RIG-I is sufficient for a robust antiviral response. Finally, by carefully quantitating the stoichiometry of stimulatory ligands, we show that the RIG-I response requires only a few cytosolic dsRNA molecules to enter the cell in order to initiate strong interferon induction. Collectively, these findings suggest that the RIG-I signaling pathway is a rapid relay that enables a sensitive antiviral response to be efficiently transmitted by a small number of activated RIG-I receptors.

## RESULTS

### Rapid signaling requires only the resident pool of RIG-I

**Figure 1.**
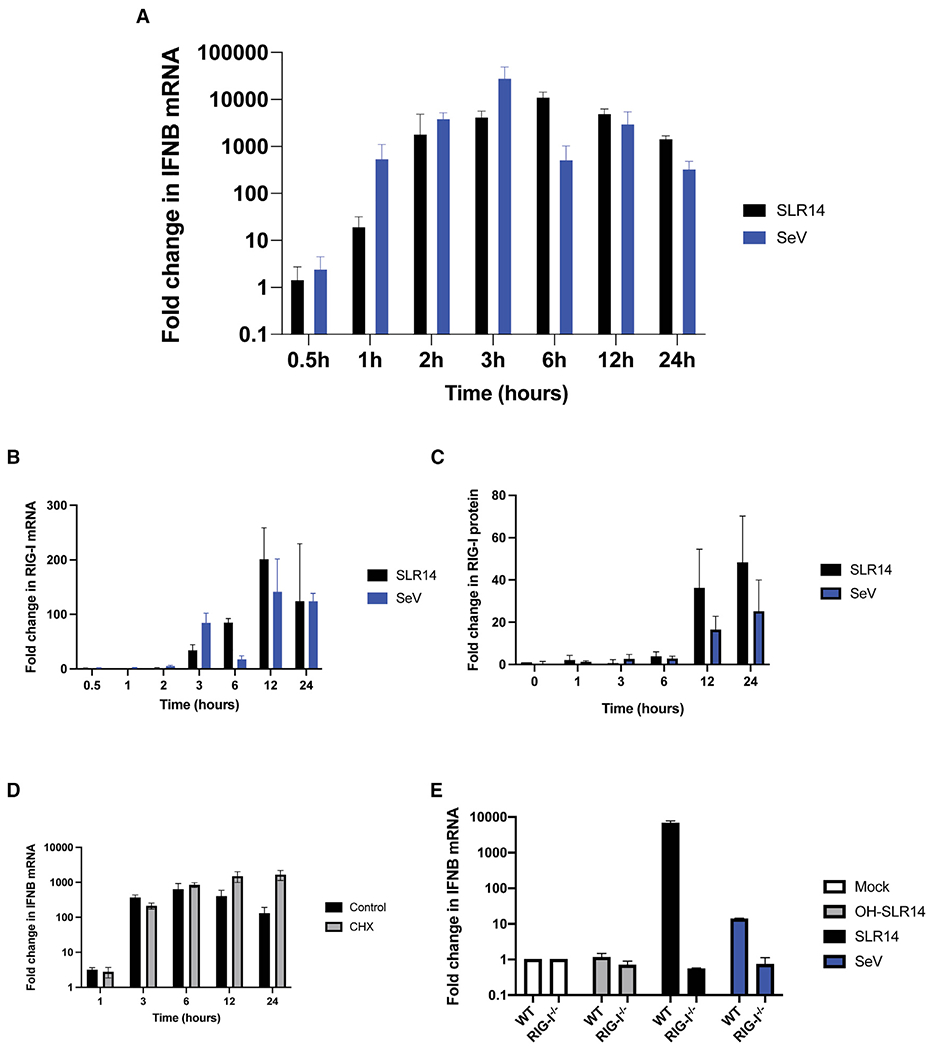
RIG-I signaling is rapid and requires only basal expression (A–C) IFNB (A) and RIG-I (B) mRNA expression and RIG-I protein expression (C) in A549 cells treated with 1 μg/mL SLR14 or 100 HA/mL SeV for 0.5–24 h. (D) IFNB mRNA expression in A549 cells pretreated with 100 μg/mL cycloheximide (CHX) 2 h prior to transfection with 1 μg/mL SLR14. (E) IFNB mRNA expression inWT and RIG-I^−/−^ A549 cells treated with 100 ng/mL SLR14, OH-SLR14, or infected with 100 HA/mL SeV for 6 h. Data are means of three independent experiments. Error bars indicate standard deviation.

We first sought to comprehensively measure endogenous RIG-I signaling kinetics over 24 h in lung epithelial (A549) cells. Cells were stimulated either via transfection of 1 μg/mL stem-loop RNA-14 (SLR14), a RIG-I-specific RNA ligand containing a single RIG-I-binding site, or with 100 haemagglutination units (HA)/mL Sendai virus (SeV), and the completion of RIG-I signaling was determined via type I interferon mRNA transcription with RT-qPCR. In all cases, regardless of RNA ligand identity or delivery method, overall signaling kinetics were strikingly rapid. SLR14 stimulated IFN-β transcription within 1 h (Figure 1A), indicating that RIG-I signaling had already been triggered before this time frame. IFN-β mRNA transcription peaked 3–6 h post-transfection and by 24 h after transfection IFNB mRNA was declining (Figure 1A). With SeV, IFN-β transcription was again initiated in less than 1 h, peaking by 3 h, and declining by 12–24 h after infection (Figure 1A). The relative decline in IFNB mRNA transcription observed at 24 h was almost 8-fold lower for SLR14-induced RIG-I signaling (fold increase of 11,500 at 6 h versus 1,500 at 24 h) and 100-fold lower for SeV-induced response than what was observed at the 3 h peak (fold increase of 29,400 at 3 h versus 343 at 24 h). These data indicate that not only is RIG-I signaling rapidly induced but also is shut off almost as quickly.

In addition to stimulating an early interferon response to intracellular dsRNA, RIG-I is itself an ISG, and this induced population of RIG-I molecules has the potential to influence the course of signaling. To monitor the correlation between kinetics of RIG-I gene expression and the kinetics of signaling, we measured both RIG-I mRNA (Figure 1B) and protein expression (Figure 1C) over time. In contrast to the kinetics displayed by RIG-I induction of type I interferon, prior to 3 h post-stimulation, there was almost no change in the levels of RIG-I mRNA or RIG-I protein during this time frame. RIG-I mRNA expression began to increase at 3 h and then rose dramatically at 12–24 h (Figure 1B) to over 100-fold higher than baseline (fold increase of 180 for SLR14 and 110 for SeV). RIG-I protein expression increased more slowly and less dramatically than mRNA expression (Figure 1C). However, even in that case, RIG-I expression continued to increase by 24 h, displaying approximately a 50-fold increase in total RIG-I protein at 24 h after challenge with SLR14. These findings have two important interpretations: first, that the rapid early burst of RIG-I signaling requires no additional RIG-I expression beyond the initial pool of receptors that is circulating in the cytosol prior to infection, and second, that the dramatic increase in RIG-I expression observed at long time points correlates with a decrease in RIG-I signaling, rather than the positive influence that was expected.

To examine whether RIG-I signaling was enhanced by interferon-induced RIG-I expression, we pretreated cells with 100 μg/mL cycloheximide for 2 h prior to SLR14 transfection to block translation and ensure that no additional RIG-I protein was expressed during the 24 h time course. Despite the inhibition of induced RIG-I translation, RIG-I signaling kinetics, as reflected in IFNB mRNA expression, did not differ in either speed or magnitude relative to untreated A549 cells during early time points (1–6 h, Figure 1D). This indicates that the resident pool of RIG-I that is present prior to infection is sufficient to produce a strong, rapid antiviral response. Interestingly, at long time points (12–24 h), cycloheximide treatment prevents IFNB mRNA expression from being turned off, as it blocks the translation of enzymes that inhibit IRF3, which is a process independent of RIG-I function.

Given the novel finding that RIG-I signaling appears to be disconnected from interferon-stimulated RIG-I expression, it was important to evaluate whether the interferon stimulation we are tracking in this study is entirely reliant on RIG-I signaling. Simultaneous treatment of wild type (WT) and RIG-I^−/−^ A549 cells with SLR14, SeV and OH-SLR14, a stem loop RNA lacking triphosphate, for 6 h demonstrated that RIG-I^−/−^ cells failed to produce any type I interferon (Figure 1E). This confirms that the signaling observed here is RIG-I-dependent and that the kinetics of this process derive entirely from action of endogenous RIG-I molecules.

### Visualization of overexpressed RIG-I reveals uniform cytoplasmic distribution during signaling

We next sought to directly observe changes in RIG-I subcellular distribution following stimulation with RIG-I-specific ligands using fluorescence confocal microscopy. First, we set out to determine whether the fluorescent tags used in this study will perturb RIG-I signaling. Attachment of a mNeonGreen or HaloTag fluorescent tag to the RIG-I amino-terminus via a flexible linker (Figure S1A) had no effect on RIG-I signaling using the IFN-β-luciferase reporter system (Figure S1B) in HEK293T cells. Specifically, HEK293T cells ectopically expressing either WT-RIG-I, mNG-RIG-I, or Halo-RIG-I produced equivalently strong interferon responses when stimulated with SLR14 containing a 5′ triphosphate and produced almost no interferon when mock-stimulated. Cells lacking the transfected RIG-I construct failed to signal in response to SLR14 treatment, indicating that the stimulated response was generated entirely by the ectopically expressed constructs within these cells. Attachment of an AlexaFluor647 fluorophore to the tetraloop region of SLR14 (Figure S1C), which is located far from the RIG-I binding site, has no effect on the stimulation of RIG-I signaling, again measured by IFN-luciferase response in HEK293T cells (Figure S1D). Therefore, for the first time, both RIG-I and a strong stimulatory RNA ligand could be monitored in live cells without disrupting the signaling pathway.

**Figure 2.**
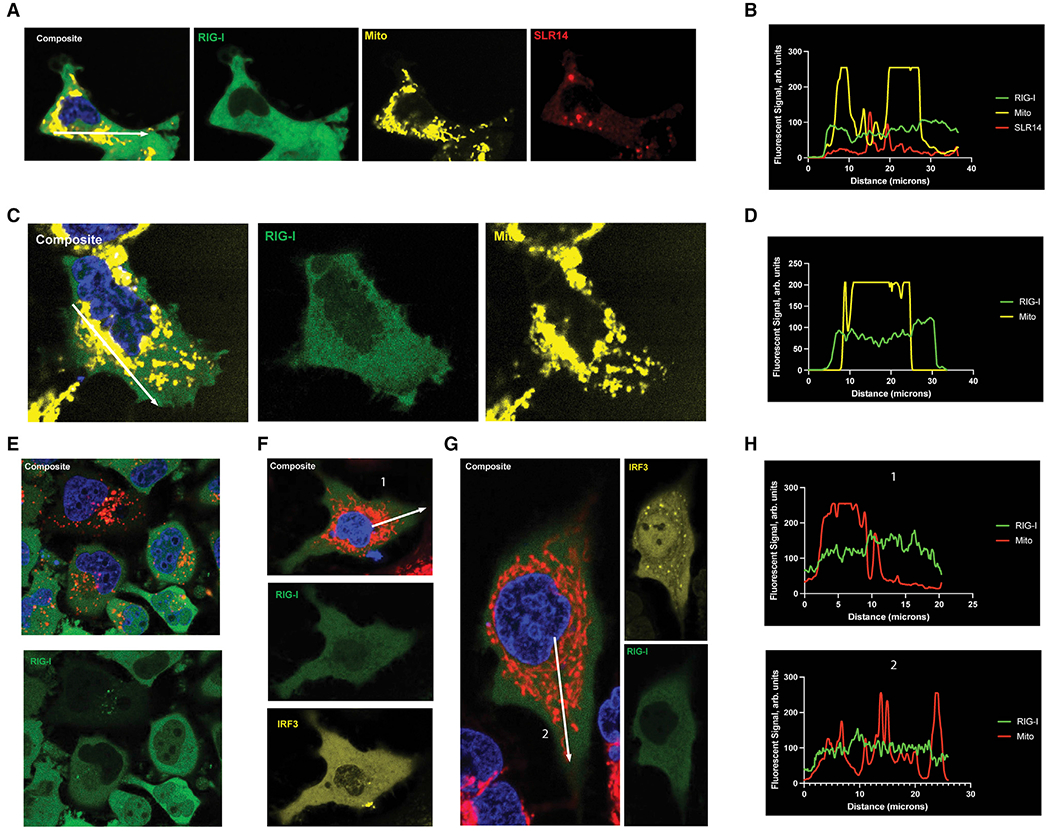
Overexpressed, fluorescently labeled RIG-I does not change distribution when stimulated (A) Representative images of HEK293T cells transfected with 300 ng/mL pUNO-Halo-RIG-I and 1 μg/mL SLR14-647 48 and 24 h prior to imaging, respectively. (B)Representative plot profile, shown as a white arrowin (A), of the fluorescent signal ofRIG-I (green), SLR14 (red), and themitochondria (yellow) across the entire cell. (C) Representative images of Huh7.5 cells transfected with 300 ng/mL pUNO-mNG-RIG-I 48 h prior to imaging and infected with 100 HA/mL SeV 24 h prior to imaging. (D) Representative line trace, drawn in (C), of the fluorescent signals of RIG-I (green) and the mitochondria (yellow) across the cell. (E) Representative images of FlpIn HeLa cells expressing Tet-inducible mNG-RIG-I treated with 0.1 μg/mL tetracycline and 1 μg/mL SLR14-647 24 and 6 h prior to imaging, respectively. (F and G) Representative images of A549 cells transfected with 300 ng/mL pUNO-mNG-RIG-I and pUNO-mCherry-IRF3, and mock-infected (F) or infected with 100 HA/mL SeV (G) 48 and 24 h prior to imaging, respectively. (H) Line traces, shown as white arrows in (F) and (G), measuring the RIG-I and mitochondrial fluorescent signal from the nucleus to the cell membrane.

Having confirmed that fluorescent labels on both RIG-I and RNA ligand do not disrupt signaling, we sought to visualize RIG-I interactions with, and responses to, a viral RNA mimic in real time with live-cell fluorescent microscopy. Additionally, labeling of the mitochondrial network with potentiometric dyes (MitoTracker-CMXRos and MitoTracker-DeepRed) enabled us to determine whether RIG-I accumulated at the mitochondrial network following RNA activation. Ectopic expression of HaloTag-RIG-I labeled with OregonGreen in HEK293T cells produced robust RIG-I fluorescence which uniformly fills the cytosol in the absence of RNA stimulation (not shown). Following transfection of 1 μg/mL of SLR14-647 for 24 h, the stimulatory RNA concentrated primarily at local cytoplasmic sites that appear as bright puncta (shown in red, Figure 2A). Despite the clear presence of the strong stimulatory RNA ligand within the cell, the subcellular distribution of RIG-I (green) remains uniform (Figure 2A). Most notably, RIG-I did not visibly accumulate at the mitochondrial network region of the cell (shown in yellow, Figure 2A), and line tracing across the cell shows that although the mitochondrial signal is punctate, RIG-I distribution remains uniform across the cell (Figure 2B). Given that HEK293T cells do not produce an interferon response without ectopic expression of RIG-I (Figure S1B), the absence of any changes in mNG-RIG-I localization was unexpected. This result was doubly surprising considering the large quantities of dsRNA delivered: 100-fold more than the minimum dose needed to stimulate an interferon response (*vide infra*).

To investigate whether this unexpected absence of any RIG-I redistribution was specific to the cell type or type of RNA used, we ectopically expressed mNG-RIG-I in Huh7.5 cells, which lack functional endogenous RIG-I expression,^28^ and then infected them with 100 HA/mL SeV (Figure 2C). Similar to our observations in HEK293T cells, introduction of viral RNA did not affect the subcellular distribution of RIG-I at 24 h (Figure 2C) in comparison to uninfected cells. In contrast to previous reports,^20^ we did not observe mass migration of RIG-I to the perinuclear region of the cell. Instead, we see RIG-I uniformly distributed through the cell, as demonstrated by line tracing (Figure 2D).

To test whether the uniform subcellular distribution of RIG-I during signaling was somehow a product of the plasmid transfection system used to express mNG-RIG-I, we incorporated the fluorescently tagged RIG-I into FlpIn HeLa cells, which express mNG-RIG-I under the control of a TetOn promoter. When these cells were stimulated with 0.1 μg/mL tetracycline and then transfected with 1 μg/mL SLR14-647 for 6 h prior to imaging (Figure 2E), the fluorescently labeled RNA (red) was clearly visible within these cells. Although some colocalization between the SLR14 puncta and RIG-I is observed, no redistribution of RIG-I to the perinuclear region of the cell was observed following stimulation.

Finally, A549 cells transiently expressing both pUNO-mNG-RIG-I (green) and pUNO-mCherry-IRF3 (yellow) were either mock infected (Figure 2F) or infected with 100 HA/mL SeV for 24 h (Figure 2G). RIG-I+/IRF3+ A549 cells showed a significant increase in IRF3 nuclear localization following SeV infection compared with mock-infected cells, indicating that RIG-I signaling was active. However, the cytosolic distribution of RIG-I remained uniform in these cells, regardless the of signaling status. This can be clearly seen again via line tracing, in which both unstimulated and actively signaling A549 cells display a uniform distribution of RIG-I throughout the cell (Figure 2H).

Throughout the time courses examined in this study (6–24 h), the distribution of overexpressed RIG-I did not change, regardless of the cell type or stimulatory RNA ligand. Despite a clear uptake of the fluorescently labeled dsRNA and direct evidence of active RIG-I signaling via IRF3, there was no large-scale translocation of RIG-I, in contradiction of previously published studies.^20^

### Live-cell imaging of early-phase RIG-I signaling shows no RIG-I accumulation at mitochondria

To determine whether the lack of apparent subcellular RIG-I redistribution was caused by the inherent limitations of ectopic RIG-I expression or whether it represents an actual property of RIG-I signaling, we used CRISPR-Cas9 to fluorescently tag the endogenous RIG-I amino-terminus with mNeonGreen in A549 cells. In this case, we used the same flexible fluorophore linker as we employed in the transiently expressed construct, and we verified that the fluorescently tagged RIG-I was expressed as effectively as WT using western blotting (Figure S2A). In addition to all of the inherent benefits of monitoring endogenously expressed genes, this approach enabled us to remove the overwhelming excess of RIG-I that is produced from transfected constructs, and it enabled us to visualize the distinct phases in endogenous RIG-I signaling observed via qPCR (Figure 1). Addition of the fluorophore on RIG-I had no impact on RIG-I stimulation by SLR14 as measured by qPCR (Figure S2B), indicating that similar to the transfected RIG-I constructs, fluorescent tagging did not disrupt or influence the RIG-I signaling response. Finally, we measured the total mean mNG signal over 24 h following stimulation by both SLR14 and SeV using flow cytometry and noted that total cellular mNG fluorescence increased significantly from 12–24 h, mirroring what was observed of total RIG-I protein expression in unlabeled cells (Figures S2C and S2D).

**Figure 3.**
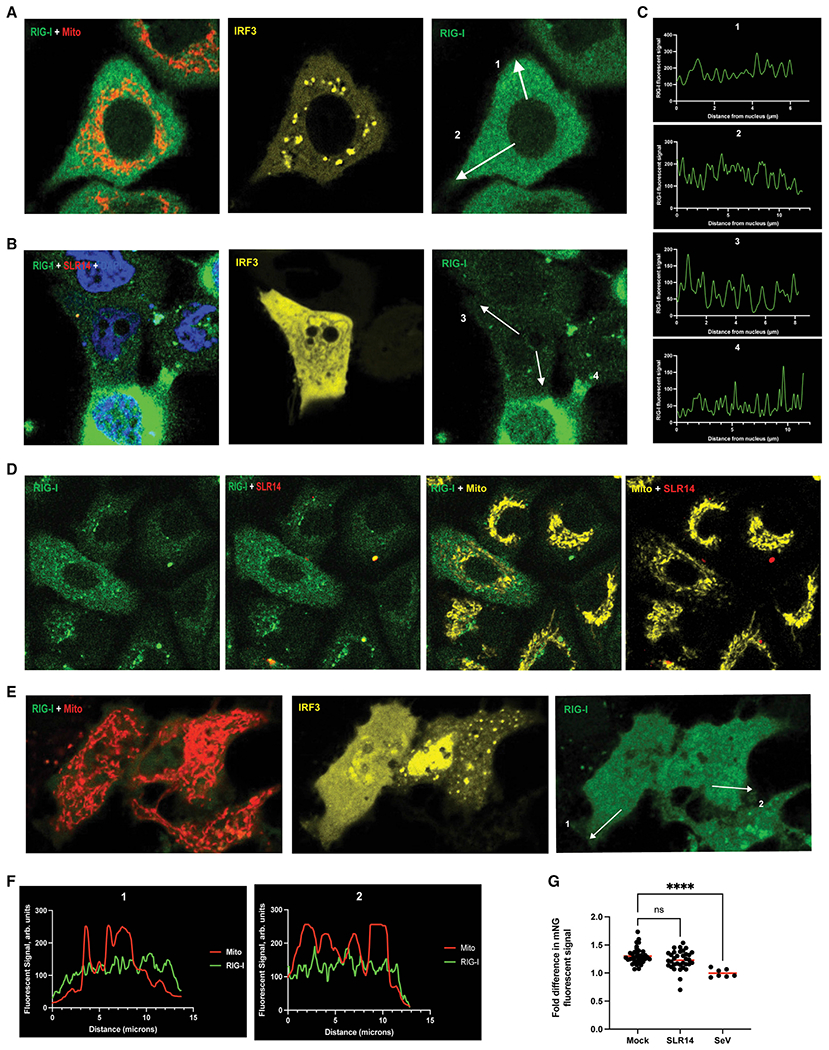
RIG-I does not aggregate at mitochondrial network during early peak phase of RIG-I signaling A549 cells expressing endogenously tagged mNG-RIG-I were visualized via confocal microscopy. (A and B) Cells transfected with 1 μg/mL pUNO-mCherry-IRF3 48 h prior to imaging, and mock-infected (A) or transfected with 1 μg/mL SLR14-647 (B) 3 h prior to imaging. (C) Representative line traces are displayed as white arrows in (A) and (B) measuring the RIG-I fluorescent signal from the nucleus to the cell membrane. (D) Cells transfected with 100 ng/mL SLR14-647 3 h prior to imaging. (E) Cells transfected with 1 μg/mL mCherry-IRF3 and 100 HA units/mL SeV 48 and 3 h prior to imaging, respectively. (F) Representative line traces are displayed as white arrows in (E) measuring the RIG-I and mitochondrial fluorescent signal from the nucleus to the cell membrane. (G) The mean RIG-I fluorescent signal colocalizing within and out (300 nm) of the mitochondrial were calculated for each cell, and the ratio of the two values was plotted for cells that were mock-infected (n = 37), SLR14-stimulated (n = 34) or SeV-infected (n = 7). Red bars represent overall mean ratio for each condition. Statistical significance performed by one-way ANOVA comparing each stimulated condition to mock-infected controls (SLR14 p = 0.0538, SeV p < 0.0001).

In the absence of dsRNA stimulation (Figure 3A), mNG-RIG-I (green) remained uniformly distributed throughout the cytosol. The relative concentration of RIG-I at the mitochondrial network (labeled with MitoTracker dye, red) was not significantly higher than in regions of the cytosol lacking mitochondria (Figure 3A). Transfected mCherry-IRF3 was completely excluded from the nucleus in the absence of stimulatory RNA (Figure 3A), confirming that signaling is inactive. These results agree with the expectation that, absent stimulation, RIG-I circulates throughout the cytosol, sampling host RNAs and remaining inactive.

At 3 h after transfection of mNG-RIG-I A549 cells with 100 ng/mL SLR14-647, the distribution of mNG-RIG-I (green) remained unchanged, with no significant redistribution to the perinuclear region, as seen by line tracing (Figure 3B). In cells ectopically expressing mCherry-IRF3 (yellow), transfection of SLR14 (red) produced a robust translocation of IRF3 to the nucleus (Figure 3B), confirming that cells transfected with SLR14 had fully activated the RIG-I signaling pathway by this early time point and confirming visually what was seen from the qPCR data (Figure 1). Measurement of the distribution of RIG-I from the nucleus to the cell membrane via line traces, depicted in (Figure 3A) and (Figure 3B) as white arrows, showed a uniform RIG-I distribution in unstimulated and actively signaling cells (Figure 3C). Stimulation of mNG-RIG-I cells with 100 ng/mL SLR14-647 (red) for 3 hand labeling the mitochondria (yellow) showed that although stimulatory RNA was present in the cells, RIG-I colocalization with mitochondria was not observed (Figure 3D).

To ensure that the absence of RIG-I redistribution following dsRNA stimulation was not specific to one dsRNA PAMP, we also visualized RIG-I distribution following infection with a high titer of SeV at 3 h post-infection. Although the viral RNA was unlabeled in this case, we were nonetheless able to simultaneously track mNG-RIG-I, mCherry-IRF3 (yellow), and the mitochondrial network (red), enabling us to measure the changes in RIG-I distribution specifically within cells where RIG-I signaling was active. At 3 h after SeV infection, RIG-I did not colocalize with the mitochondria, although in these same cells, IRF3 (yellow) had translocated to the nucleus, thereby indicating that RIG-I signaling was completed (Figure 3E). At 3 h post-infection, line tracing from the nucleus to the cell membrane again indicated that RIG-I did not accumulate at the perinuclear region, despite the fact that RIG-I signaling was active. Therefore, accumulation of RIG-I at the mitochondrial network during active signaling at 3 h was not observed, regardless of the stimulatory RNA ligand introduced to the cell.

To quantitatively analyze any RIG-I-MAVS colocalization, we measured the mean mNG-RIG-I fluorescent signal at the position of the mitochondria and compared it with the mean fluorescent mNG-RIG-I signal in cytosolic regions outside of the mitochondria. The ratio of these values allowed for direct comparison of the mNG-RIG-I cellular distribution between different conditions. Compared with mock-infected cells, stimulation with SLR14-647 at 3 h did not increase RIG-I colocalization with the mitochondria (p = 0.09) and stimulation with SeV at 3 h resulted in less RIG-I colocalization with the mitochondria relative to mock (p < 0.0001) (Figure 3G). Therefore, during the time frame when RIG-I signaling is strongest, no increase in RIG-I concentration at the mitochondria is observed.

In our immunofluorescence experiments, activation of RIG-I signaling and subsequent IRF3 translocation into the nucleus was not accompanied by a detectable accumulation of mNG-RIG-I at the mitochondrial network 3 h post-stimulation. Therefore, our data suggest that mass-translocation and aggregation are not required during the peak phase of RIG-I signaling.

### Late-phase RIG-I stimulation involves increased expression without mitochondrial accumulation

Although RIG-I did not accumulate at the mitochondrial network during the peak phase of RIG-I signaling (3 h), we sought to determine whether this accumulation might instead occur at later times after RIG-I stimulation with viral RNA (vRNA) PAMPs (~24 h), in keeping with results from previous studies conducted at late time points. Prior studies of RIG-I subcellular organization during active signaling had reported RIG-I redistribution 10–40 h after infection,^19–21^ and it was therefore of interest to replicate these results.

**Figure 4.**
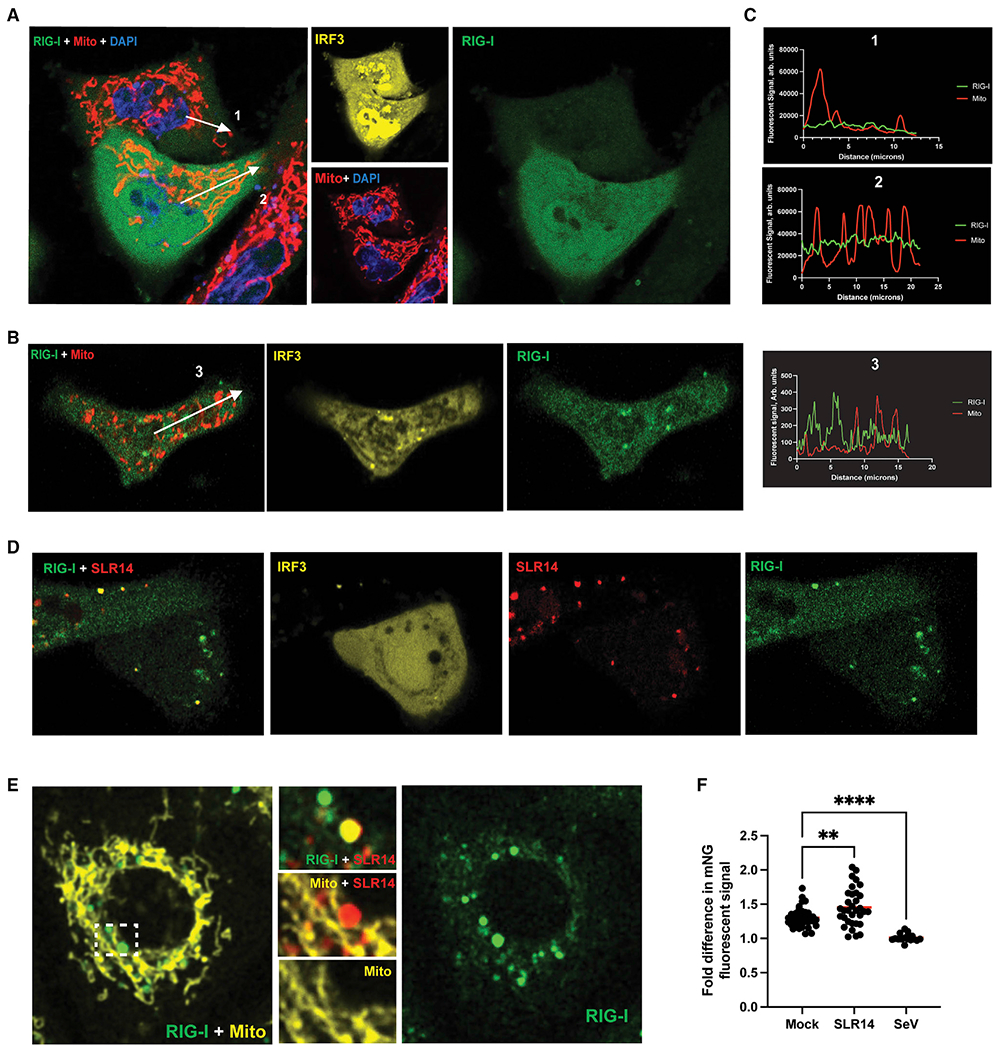
RIG-I does not form massive oligomers at the mitochondrial network during late phase of RIG-I signaling A549 cells expressing endogenously tagged mNG-RIG-I were visualized via confocal microscopy. (A and B) Cells transfected with 1 μg/mL pUNOmCherry-IRF3 48 h prior to imaging, stimulated with 1 μg/mL SLR14 (A) and 100 HA units/mL SeV (B) 12 and 24 h prior to imaging, respectively. (C) Representative line traces displayed as white arrows in (A) and (B) measuring the RIG-I and mitochondrial fluorescent signal from the nucleus to the cell membrane. (D) Cells transfected with 1 μg/mL pUNOmCherry-IRF3 and 1 μg/mL SLR14-647 48 and 24 h prior to imaging, respectively. (E) Cells transfected with 100 ng/mL SLR14-647 24 h prior to imaging. (F) The mean RIG-I fluorescent signal directly overlapping and 300 nm away from the mitochondrial network were calculated for each cell, and the ratio of the two values was plotted for cells that were mock-infected (n = 37), SLR14-stimulated (n = 33) or SeV-infected (n = 17). Red bars represent overall mean ratio for each condition. Statistical significance performed by one-way ANOVA comparing each stimulated condition to mock-infected controls (SLR14 p = 0.0045, SeV p < 0.0001).

At 12 h post-transfection with 1 μg/mL unlabeled SLR14, mNG-A549 cells continued to actively signal, as seen by the elevated nuclear levels of IRF3 (yellow); however, the distribution of RIG-I remained unchanged (Figure 4A). Comparison of the composite image of RIG-I (green) and mitochondria (red) to RIG-I alone showed no significant evidence of RIG-I accumulation at the mitochondria (Figure 4A). To determine whether the absence of RIG-I redistribution was simply a property of SLR14, we also examined the distribution of mNG-RIG-I at 24 h after SeV infection (100 HA/mL) (Figure 4B). We observed nuclear translocation of mCherry-IRF3 (yellow) within these cells, indicating that although RIG-I signaling is declining overall during the later stages of infection, downstream stages of active RIG-I signaling can still be seen within these cells. However, the stimulation of RIG-I with SeV for 24 h does not increase colocalization of mNG-RIG-I with the mitochondria (red). Line traces of the overall distribution of RIG-I within both SLR14-transfected and SeV-infected cells showed a uniform distribution of RIG-I throughout the cytosol (Figure 4C).

During late time points, after cells had been transfected with labeled SLR14, we continued to observe a similar RIG-I subcellular distribution as observed at 3 h. At 24 h post-transfection with 100 ng/mL SLR14-647, the distribution of RIG-I remained uniform, whereas signaling remained active, as seen via IRF3 translocation to the nucleus (Figure 4D). As observed at earlier time points, RIG-I did not accumulate along the mitochondrial network (yellow) after stimulation with SLR14-647 (red) (Figure 4E), rather RIG-I and SLR14 colocalized in regions distinct from the mitochondria.

To ensure that the lack of RIG-I colocalization with mitochondria was not limited to these specifically chosen images, we measured the total mNG-RIG-I fluorescent signal at the position of the mitochondria and compared it with the fluorescent mNG-RIG-I signal in cytosolic regions outside of the mitochondria under various conditions, using multiple samples to obtain a good statistical sampling. Compared with mock-infected cells, stimulation with SLR14-647 at 24 h resulted in a small increase in RIG-I colocalization with the mitochondria (p = 0.003), whereas stimulation with SeV at 24 h resulted in less RIG-I colocalization with mitochondria relative to mock (p < 0.0001) (Figure 4F).

To determine whether the use of mitochondrial labeling was an adequate proxy for the location of MAVS, we then compared the distribution of RIG-I with immunolabeled MAVS in fixed cells in order to determine whether the degree of RIG-I colocalization had changed. Fixed mNG continued to fluoresce, enabling continued visualization of mNG-RIG-I, and mock-treated cells showed a similar diffuse cytosolic distribution of mNG-RIG-I and mCherry-IRF3 as observed in the live-cell images (Figure S3A). At 3 h after infection with 100 HA/mL SeV, fixed mNG-RIG-I showed no increase in colocalization with immunolabeled MAVS (Figure S3B). Similarly, at 24 h after infection with 100 HA/mL SeV, fixed mNG-RIG-I showed colocalization with immunolabeled MAVS (Figure S3C). We quantified the degree of RIG-I colocalization with MAVS by measuring the total mean cellular mNG-RIG-I fluorescent signal in MAVS+ regions of the cell compared with outside MAVS+ regions. Compared with mock-infected cells, stimulation with SeV at 3 h resulted in a decrease in RIG-I colocalization with MAVS (p = 0.02), whereas stimulation with SeV at 24 h resulted in no change in RIG-I colocalization with MAVS (p = 0.23) (Figure S3D). Furthermore, to confirm that RIG-I distribution was not altered by addition of the mNeonGreen tag, we immunolabeled fixed mNG-RIG-I A549 cells for total endogenous RIG-I. Upon stimulation with 100 HA/mL SeV for 3 h, mNG-RIG-I (green) and immunolabled RIG-I (yellow) exhibited similar subcellular distributions and showed no increased colocalization with immunolabeled MAVS ([red] Figure S3E). This indicates that unlabeled RIG-I displays the same overall pattern during active signaling as labeled RIG-I and that the fluorescent tags did not block the formation of RIG-I oligomers.

### Colocalized accumulations of RNA and RIG-I can be seen, but they are not functionally related to signaling

Although endogenous RIG-I did not redistribute toward the mitochondria, some of the RIG-I pool was observed to form puncta that colocalized with transfected RNA throughout the time course of signaling. To determine the kinetics of the appearance of these RIG-I:RNA bodies, we quantified RIG-I puncta at both early and late time points.

**Figure 5.**
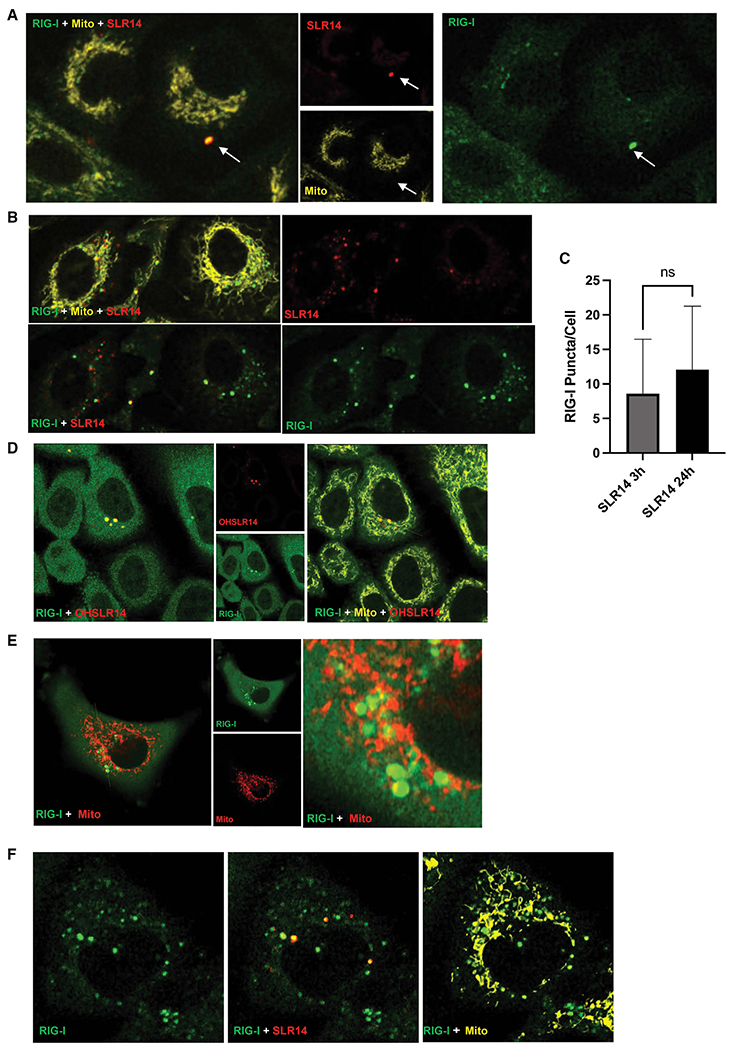
RIG-I:SLR RNPs accumulate over time and are connected to RIG-I expression rather than signaling A549 cells expressing endogenously tagged mNG-RIG-I were visualized via confocal microscopy. (A and B) Cells transfected with 100 ng/mL SLR14-647 3 h (A) and 24 h (B) prior to imaging. (C) Quantification of the mean number of RIG-I bodies in each cell at 3 h (A, n = 42 cells) and 24 h (B, n = 31 cells) (p = 0.0873). (D) Cells transfected with 1 μg/mL OH-SLR14-647 24 h prior to imaging. (E) Cells treated with 100 U/mL IFN-α 24 h prior to imaging. (F) Cells treated with 100 μg/mL cycloheximide 2 h prior to transfection with 100 ng/mL SLR14-647 for 24 h prior to imaging.

Although mNG-RIG-I (green) did not colocalize with the mitochondria at either 3 or 24 h post-transfection with 100 ng/mL SLR14-647, RIG-I did appear to form detectable cytosolic bodies in other, non-mitochondrial regions of the cytosol (Figure 5A). Furthermore, many of these RIG-I bodies colocalized with SLR14-647 (red), suggesting that these RIG-I bodies also contained dsRNA (Figure 5A). Continued visualization of RIG-I bodies at 24 h after transfection with 100 ng/mL SLR14-647 showed that colocalization with dsRNA continued (Figure 5B) and that the total number of RIG-I bodies increased, although the increase in mean RIG-I bodies/cell from 3 to 24 h was not statistically significant (Figure 5C).

To determine whether these RIG-I:RNA bodies are connected to signaling, we transfected mNG-RIG-I A549 cells with OH-SLR14-647, which binds RIG-I with relatively high affinity, but lacks 5′ phosphates and cannot stimulate a signaling response.^8^ At 3 h, OH-SLR14 (red) aggregated in similar puncta within cells, and RIG-I puncta (green) colocalized to similar degree (Figure 5D).

Because RIG-I puncta are most prevalent at late times after RNA transfection, and they associate with any type of dsRNA, regardless of whether downstream IFN signal can be activated, it is possible that these bodies have a distinct function that is related to RIG-I as an ISG. To evaluate this possibility, we directly stimulated mNG-RIG-I A549 cells with 100 U/mL IFN-α for 24 h (in the absence of stimulatory RNA) and observed continued formation of RIG-I aggregations and puncta (Figure 5E), indicating that aggregated RIG-I puncta are not dependent on dsRNA ligands but instead form directly in response to interferon stimulation.

Given that RIG-I aggregation at late times may represent a secondary, non-signaling response to viral infection, we sought to determine if the basal pool of cytoplasmic RIG-I is sufficient to induce formation of these structures. To this end, we used cycloheximide to block downstream, interferon-induced translation of additional RIG-I receptors. Treatment of cells with 100 μg/mL of cycloheximide for 2 h prior to dsRNA stimulation restricted the cellular pool of RIG-I exclusively to the receptors present before viral RNA stimulation, so that by 24 h after transfection with 100 ng/mL SLR14-647, the overall cytosolic level of RIG-I decreased significantly (Figure 5F). However, although RIG-I bodies were smaller in size, they continued to form and colocalize with labeled SLR14-647 (Figure 5F), indicating that basal RIG-I populations can form these non-signaling cytosolic bodies.

### RIG-I signaling can be triggered by cytosolic entry of only a few viral RNAs

Given the rapid signal produced by RIG-I in response to viral RNA and the absence of observable RIG-I complexes at the mitochondrial network during RIG-I signaling, we wondered whether large amounts of viral RNA are actually required to mount this response, as suggested by the prevailing model for RIG-I signaling. To explore the required RNA stoichiometry using an orthogonal approach, we quantified the number of RIG-I ligands required to stimulate RIG-I signaling.

**Figure 6.**
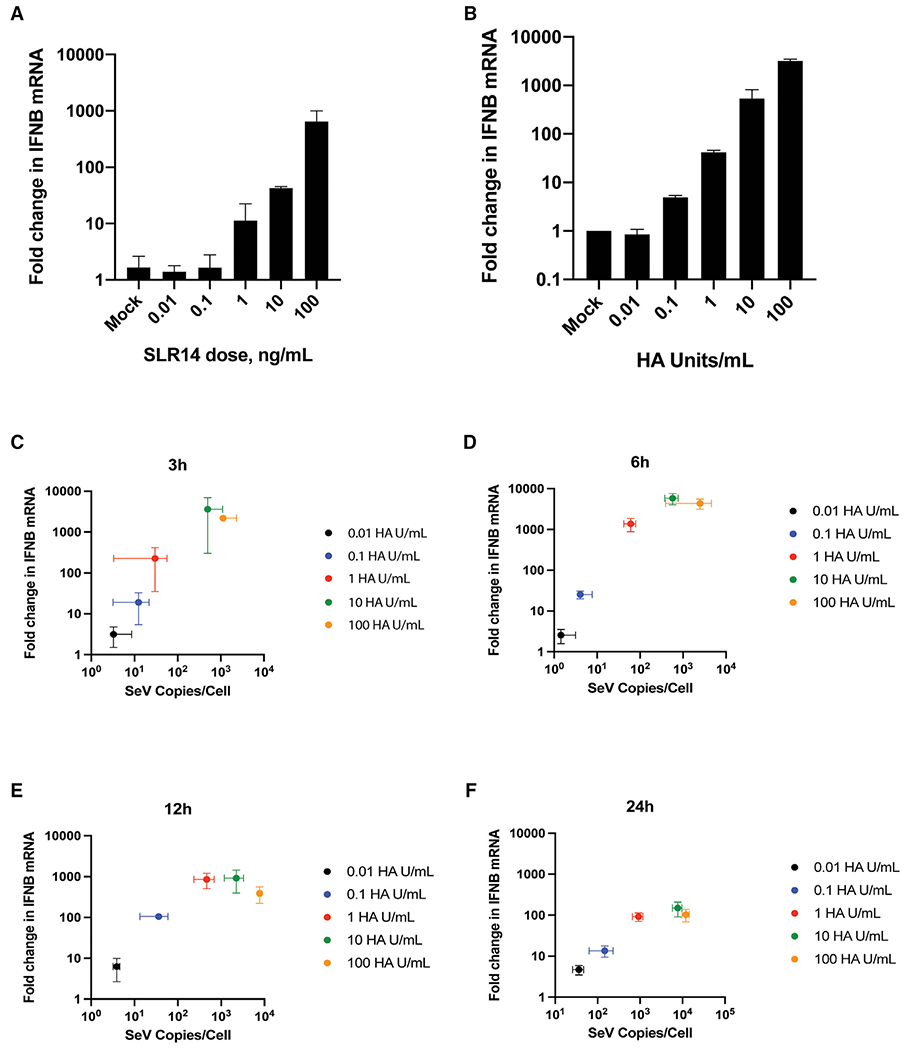
Quantification of RIG-I ligands reveals RIG-I signaling to be highly efficient. (A and B) IFNB mRNA expression in A549 cells transfected with SLR14 (A) or SeV (B) at 10-fold dilutions for 6 h. (C–F) IFNB mRNA expression in A549 cells infected with 0.01–100 HA/mL SeV for 3–24 h. Total DI copies/cell were plotted against fold change in IFNB mRNA expression to determine the total number of RNAs required to stimulate an IFN response at 3 h (C), 6 h (D), 12 h (E) and 24 h (F). Data are means of three independent experiments. Error bars indicate standard deviation.

As an initial approach for determining the response of RIG-I to varying concentrations of viral RNA, we transfected A549 cells with 0.01 to 100 ng/mL SLR14 for 6 h (Figure 6A). We observe that RIG-I signaling is initiated by 1 ng/mL SLR14 and continues to increase to 100 ng/mL. Similarly, infection of A549 cells with 10-fold dilutions of SeV for 6 h produced a response beginning at 0.1 HA/mL, with continued increase in RIG-I signaling up to a 1,000-fold increase in viral RNA (Figure 6B). The discrete rise in RIG-I signaling that is observed with both types of RNA indicates that although the highly sensitive RIG-I receptor is capable of mounting an antiviral response upon exposure to minute quantities of viral RNA, a significant pool of inactive RIG-I remains available for producing a stronger stimulatory response to a more substantial dose of virus. Additionally, both minimum stimulatory doses are 100- to 1,000-fold lower than the doses used to visualize RIG-I *in cellulo* in both overexpressed and endogenous imaging experiments. Therefore, despite stimulation with a quantity of vRNA sufficient to saturate the RIG-I signaling response, the total lack of RIG-I redistribution indicates that active RIG-I signaling is again either too minute or too transient to be observed.

The amount of RNA required to stimulate RIG-I signaling was quantitated using SeV defective interfering (DI) RNA (STAR Methods). We found that approximately an average of 10 SeV DI RNA copies/cell was sufficient to initiate a RIG-I signaling response at 3 or 6 h (Figures 6C and 6D). Additionally, although SeV DI copies/cell kept increasing throughout the 24 h time course, the fold increase of IFNB mRNA already peaked at 6 h and declined thereafter (Figures 6C–6F). Although the total number of RNA ligands increases over time as viruses replicate, RIG-I signaling displays an initial burst of interferon and then declines over time. Altogether, these findings suggest that the RIG-I signaling response is highly sensitive. RIG-I does not require vast quantities of viral RNA as a template for the formation of massive oligomeric signaling complexes. Rather, RIG-I binding to a small handful of viral RNAs is sufficient to quickly initiate a strong antiviral response.

## DISCUSSION

In this study, we directly monitored the kinetic behavior and spatial location of activated RIG-I in living cells. We find that RIG-I responds remarkably rapidly upon stimulation with RNA PAMPs and that the receptor selectively identifies, binds, and transmits a robust transcriptional response in less than an hour. The speed of this response is sufficiently rapid to match the fast kinetics of viral infection and replication, which must be detected immediately to marshal appropriate cellular defensive responses. Signaling processes that occur after 24 h would be too slow to provide an effective antiviral defense, and however, previous studies visualizing the distribution of RIG-I have focused almost exclusively on this late time frame, after which the initial burst of functional signaling has long since passed. It was therefore important to take a fresh look at the RNA ligand requirements and subcellular localization kinetics of the RIG-I receptor during the course of signal induction.

An examination of RNA ligand stoichiometry revealed that RIG-I signaling requires tiny quantities of RNA PAMPs. We found that the entire process can be triggered by as few as an average of 10 molecules of intracellular viral, in broad agreement with prior measures of RIG-I sensitivity.^16,30^ Like the rapid speed of RIG-I activation, this behavior is consistent with a signaling pathway that must be triggered immediately and efficiently upon viral infection. Our findings similarly align with studies showing that incoming or replication-defective viral RNAs are still capable of initiating a robust RIG-I-specific interferon response.^17,29^

The fact that antiviral signaling by RIG-I is rapid and highly efficient is consistent with the fact that this process is driven by the resident pool of constitutively expressed RIG-I and that it does not involve additional rounds of RIG-I expression, signal amplification, or engagement of RIG-I as a downstream ISG. Specifically, when cycloheximide is used to block downstream RIG-I translation, one still observes robust RIG-I signaling upon treatment with viral RNA PAMPS, occurring with essentially the same magnitude and kinetics as in the absence of cycloheximide. Again, this makes sense given that the antiviral response must be immediate, and a requirement for additional receptor expression would slow down a critical first-response in the cell.

As reported in previous studies,^27^ we observe that RIG-I is both a trigger of interferon expression and an ISG and that RIG-I protein expression increases after cellular stimulation with RNA PAMPs. However, this rise in protein expression occurs many hours after RNA stimulation, and it correlates with a decline in overall IFN signaling. This finding raises a larger and even more intriguing question: If boosted levels of RIG-I expression do not enhance antiviral signaling, then does the overexpression of RIG-I serve as some other type of antiviral effector? There are several possible models for this, and they are best addressed by monitoring the subcellular localization of RIG-I *(vide infra).*

The fact that RIG-I signaling is so fast, requires so few copies of viral RNA, and is fully activated by short dsRNA molecules is inconsistent with conventional models for RIG-I signaling. This is understandable, as earlier models for the pathway were based on experiments conducted long before advances in structural biology, and cellular imaging made it possible to visualize stages of the process. According to the prevailing “mass aggregate” model for RIG-I signaling, viral RNA stimulation causes RIG-I to accumulate, polymerize, and form massive filaments on long regions of double-stranded viral RNA, thereby presenting a large array of free CARDs for interaction with the MAVS adapter protein.^31^ Recent studies have even proposed that ubiquitination strengthens RIG-I signaling by bridging large RIG-I:RNA filaments and thereby increasing the size of signaling complexes.^32^ This model originated from *in vitro* studies in which high concentrations of isolated CARD protein domains were shown to aggregate in buffer solution, resulting in a behavior that was taken as proxy for signaling. Because isolated CARDs form aggregated polymers *in vitro* and because the mutations that disrupted this CARD polymerization in a test tube were also observed to inhibit RIG-I signaling in cells,^22^ it was extrapolated that large aggregates of full-length RIG-I are necessarily required for signaling *in cellulo* and that aggregates are markers for RIG-I activation. Furthermore, according to this model, increased RIG-I expression, stimulated by interferon, would be expected to strengthen RIG-I signaling by adding to the size of the RNA:RIG-I mass aggregate. However, published attempts to visualize RIG-I oligomerization, and that of other components of the pathway, directly in cells have failed.^33^ For example, split-luciferase complementation studies failed to detect any increase in RIG-I complex formation at 4 h post-transfection with poly(I:C).^34^ Similarly, our observations on the speed and efficiency of RIG-I signaling support a dramatically different mechanistic model for RIG-I signaling, suggesting that it requires only a small handful of active receptors to relay a fully active signal to MAVS and thereby transmit the antiviral signaling response further downstream. Given that conventional models of RIG-I signaling posit the obligate accumulation of RIG-I protein on the mitochondrial membrane, we set out to monitor the dynamics of RIG-I expression and subcellular localization directly.

Using CRISPR-tagged endogenous RIG-I, imaging studies revealed a uniform cytosolic distribution of the receptor, confirming that RIG-I circulates continuously throughout the cytoplasm. Similar results in a broad array of cell lines were observed with tagged, transfected RIG-I. After transfection with RNA PAMPs, RIG-I displays the same uniform cytoplasmic distribution during the most robust stage of signaling, which is inconsistent with the requirement for a massive aggregated signaling complex. During these same early time frames after RNA induction, we confirmed that dsRNA successfully entered the cell, transcription factor IRF3 had translocated to the nucleus and that interferon was expressed, thereby confirming that the RIG-I signaling pathway was fully functional and that we were observing an accurate picture of RIG-I behavior during active signaling. In addition there were no large changes to the subcellular organization of infected cells. Rather, our results suggested that active signaling complexes, such as those involved in a rapid relay from a handful of active receptors are beneath the detection limit for visualization by fluorescence confocal microscopy. It is important to note that the absence of RIG-I colocalization with MAVS does not suggest a MAVS-independent signaling process, which is well supported in the literature. Given that both stem loop RNA^8,35^ and SeV^11,13,36^ stimulation of interferon are dependent on MAVS *in cellulo* and *in vivo,* the signaling events described in this work are expected to require the RIG-I:MAVS interaction.

It is important to note that large, aggregated deposits of RIG-I were never observed at the mitochondrial network, whether measured early or late in the process, in direct contradiction of previous studies suggesting aggregation at either the mitochondria or the perinuclear region.^20,21,37,38^ However, again, this is likely due to differences in the methods employed during earlier studies. Given the increase in total RIG-I ISG expression that occurs after RNA stimulation, RIG-I concentration is likely to be elevated at the mitochondria, just as it is elevated everywhere else in the cell.

At all time frames after RNA transfection, but especially during later stages (~24 h), cellular puncta containing abundant RIG-I protein accumulate at localized sites in the cell. In certain cases, these RIG-I species co-aggregated with transfected RNA, regardless of whether the RNA was a stimulatory PAMP. Importantly, these RIG-I:RNA bodies, which do not associate with the mitochondrial network, also form after direct treatment with IFN-α (in the absence of transfected RNA), indicating that their formation is independent of RNA identity and is linked to the role of RIG-I as an ISG. These RIG-I RNP bodies are reminiscent of RIG-I-containing “antiviral stress granules” that have been previously reported.^19^ These species contained not only RIG-I and influenza A virus (IAV) RNA but also other markers of stress granules, and they also appeared during the latter phases of infection.^19^ Importantly, the stress granules reported previously were observed only after 12 h post-infection, suggesting that they form too late to be linked with productive antiviral signaling. Indeed, our observation of RIG-I colocalization with both signaling and non-signaling dsRNAs suggests that these puncta of accumulated RIG-I are not related to signaling at all. These RIG-I-containing bodies may simply represent locations where RIG-I accumulates, post-signaling or otherwise, as it awaits degradation. More interesting, however, is the possibility that these RIG-I bodies serve a non-signaling effector function, perhaps by sequestering viral (and also host) RNAs in non-membranous subcellular structures where it cannot be replicated or translated.

In summary, we have determined that RIG-I signaling is quickly initiated from a small number of dsRNAs in the cytosol and that it requires only the constitutively expressed level of RIG-I receptors. Even when large amounts of viral RNA are present, we observe that RIG-I signaling proceeds without aggregation at the mitochondrial network. This suggests that the active RIG-I signal is transmitted transiently, rather than accumulating as a massive oligomeric complex at the MAVS interface. Finally, we directly observe that RIG-I forms large, cytosolic RNP bodies with dsRNA, regardless of the RNA identity or size, suggesting that RIG-I aggregation is connected to the formation of specialized RNP bodies rather than with signaling. We believe that these findings help to distinguish RIG-I signaling from RIG-I oligomerization, showing that these are separate phenomena, thereby enabling future studies to better understand the mechanistic role of RIG-I during the cellular antiviral response.

### Limitations of the study

Although this study indicates that certain aspects of the standard RIG-I signaling model require revision, it is not a comprehensive dissection of the entire signaling cascade and all the macromolecular complexes that participate along the pathway. For example, we used endogenously tagged RIG-I to monitor its spatial and temporal behavior during active signaling, but our parallel studies of MAVS and IRF-3 depended on immunolabeling studies of fixed samples, which ultimately limits the conclusions that we can draw about the spatiotemporal dynamics of these important components of the signaling pathway. More thorough live-cell imaging and complementary approaches, such as FRAP (fluorescence recovery after photobleaching), are required for a complete picture of the overall pathway, but these will necessitate a comprehensive endogenous labeling approach that includes tags on MAVS and Riplet. A second limitation is that although our data suggest that massive RIG-I-MAVS oligomers do not contribute to active signaling, our conclusions are restricted by the limited spatial resolution of the methods employed here (about 140 nm laterally and 400 nm axially). Similarly, although endogenous RIG-I-MAVS colocalization was not detectable during active signaling in this study, this may be explained by a relatively transient RIG-I-MAVS interaction. Implementation of endogenous split-reporter systems may ultimately be helpful for investigating such interactions.

## STAR★METHODS

### RESOURCE AVAILABILITY

#### Lead contact

Further information and requests for resources and reagents should be directed to and will be fulfilled by the lead contact, Anna Pyle (anna.pyle@yale.edu).

#### Materials availability

All plasmid constructs described in this paper and the mNG-RIG-I A549 cells are available upon request, pending material transfer agreement.

#### Data and code availability

All data reported in this paper will be shared by the lead contact upon request.

This paper does not report original code.

Any additional information required to reanalyze the data reported in this paper is available from the lead contact upon request.

### EXPERIMENTAL MODEL AND SUBJECT DETAILS

#### Cell lines

A549 cells, including CRISPR-generated knock-in cell lines and RIG-I^−/−^ cells, were maintained in Ham’s F-12 medium containing 10% fetal bovine serum and 100 U/mL penicillin/streptomycin. HEK293T, Huh7.5 and HeLa cell lines were maintained at 37C in Dulbecco’s modified eagle medium (DMEM) containing 10% fetal bovine serum and 100 U/mL penicillin/streptomycin. All cells maintained, and all experiments and imaging carried out, at 37C in 5% CO_2_.

### METHOD DETAILS

#### RNA synthesis, purification, and modification

Stem Loop RNAs (5’ GGAUCGAUCGAUCG UUCG CGAUCGAUCGAUCC 3’) were synthesized on a MerMade 12 DNA-RNA synthesizer using previously described methods^43^ with either a triphosphate or hydroxyl 5’ guanosine. Synthesized RNAs were deprotected and purified via polyacrylamide gel and analyzed via mass spectrometry for purity. AlexaFluor-647 NHS ester (ThermoFisher) was conjugated to an amino-modified uridine (C6) (ChemGenes) on the loop region (5’ GGAUCGAUCGAUCG U**U**CG CGAUCGAUCGAUCC 3’).

#### SLR14 transfection and SeV infection

SLR14 was added to OptiMEM (ThermoFisher) at 10X indicated concentrations using 20 μL/mL RNAiMAX (Invitrogen), and the RNA/lipid mixture was transfected onto cells at 1/10 total media volume. 2000 HA/mL stocks of Cantell strain Sendai virus (Charles River) were thawed, vortexed, and diluted in OptiMEM to the corresponding doses, then added to cells at 37°C for 60 min before replacement with fresh media.

#### RT-qPCR

Following RNA transfection/viral infection, total cellular RNA was extracted via column purification (Omega Biotek) and treated with Turbo DNase (ThermoFisher) to remove DNA contamination. cDNA was reverse-transcribed from 500 ng total RNA extract with Superscript III (ThermoFisher) following manufacturer’s protocols using random hexamer primers (ThermoFisher). Following reverse-transcription RNA was degraded with 333 mM NaOH and cDNA purified via ethanol precipitation. RT-qPCR was performed using LightCycler 480 Mastermix (Roche) with the following primers: IFNB [Fwd: GCGACACTGTTCGTGTTGTC, Rev: GCCTCCCATT CAATTGCCAC] and RIG-I [Fwd: CTGATTGCCACCTCAGTTGC Rev: GTCCCATGTCTGAAGGCGTA] were normalized to ACTB [Fwd: TTCCAGCAGATGTGGATCAG Rev: GGTGTAACGCAACTAAGTCA], and further normalized to Mock-infected wells to determine fold induction of gene expression.

#### Western Blotting

Protein lysates were extracted from A549 cells treated with 1 μg/mL SLR14 or 100 HA/mL SeV for 1h to 24h, the expression of both RIG-I and GAPDH was measured via western blot, and the difference in RIG-I expression was calculated relative to mock-infected wells. Cells lysed at indicated times with NP-40 buffer containing protease and phosphatase inhibitors (Roche). Equal quantities of lysate were separated on 4-20% SDS-PAGE (Bio-Rad) and transferred onto 0.45 μm^2^ PVDF membrane, then blocked with TBST + 5% milk before treatment with anti-RIG-I (Cell Signaling Technology) and anti-GAPDH antibodies overnight at 4C. Following washing, blots were treated with HRP-conjugated secondary antibodies and visualized using a blot imager (Bio-Rad). Blots were background-subtracted and the total band intensity was measured via ImageJ, then the ratio of RIG-I/GAPDH expression calculated from both bands.

#### Immunofluorescence

HEK293T, WT A549 and mNG-RIG-I A549 cells were seeded at 1 x 10^5^ cells/mL on 18mm acid-washed #1.5 glass coverslips in 1mL media and transfected for the indicated timepoints. HaloTag-RIG-I was labeled with 1 μM HaloTag OregonGreen for 15 min prior to fixation. Mitochondria were labeled with 100 nM MitoTracker-CMXRos or MitoTracker-DeepRed for 30 min prior to fixation. Cells were fixed with 4% paraformaldehyde for 15 min at room temperature. Cells were permeabilized with PBS + 0.1% Triton X-100 for 10 min, then washed and blocked with PBS + 3% BSA and 5% normal donkey serum for 20 min. Cells were then treated overnight at 4C with RIG-I and/or MAVS antibodies at 1:100 dilution in PBS + 3% BSA, washed five times in PBS + 3% BSA, and then treated with AlexaFluor-conjugated secondary antibodies at 1:500 dilution in PBS + 3% BSA for 30 minutes at RT. Cells were again washed 5 times, then mounted onto glass slides with Fluormount G + DAPI.

#### mNG-RIG-I CRISPR Knock-in

RIG-I-targeted sgRNAs (guide: TTGCAGGCTGCGTCGCTGCT, IDT) and 3 μg HDR templates were electroporated into A549 cells and expanded for 7-10 days. Cells were then treated with SLR14 at 1 μg/mL for 16h, then FITC+ cells were sorted via FACS and clonally expanded. Successful mNG+ knock-ins were screened via Sanger sequencing and immunoblot. Successful knock-in cells were examined for effective RIG-I signaling via qPCR and increased expression via flow cytometry.

#### Flow Cytometry

mNG-RIG-I A549 cells were stimulated with 1 μg/mL SLR14-647, 100 HA/mL SeV, or mock-stimulated for 3 to 24h, then trypsinized, pelleted and resuspended in PBS + 0.1% BSA, then the FITC fluorescent signal in 10,000 live cells for each condition was measured on a BD FACS LSR Fortessa X20 (Science Hill Flow Cytometry Facility). The mean cellular FITC signal and standard deviation for each condition were calculated via FlowJo.

#### Live Cell Confocal Fluorescence Microscopy

mNG-RIG-I cells were seeded on 35 mm dishes at 100,000 cells/mL in 2 mL F-12k. 48h prior to imaging, cells were transfected with 200 μL plasmid solution (pUNO-mCherry-IRF3 prepared in OptiMEM at 2.5 μg/mL with 40 μL/mL Lipofectamine 2000). Then, at 3-24h prior to imaging, cells were transfected with 100 ng/mL SLR14-647 or infected with 100 HA/mL SeV. Images were collected on an LSM 880 Airyscan confocal microscope (Zeiss) with 63X PlanApo lens. Image processing was carried out in FIJI.

#### RIG-I Mitochondrial and MAVS colocalization

For analysis of RIG-I colocalization with mitochondria or MAVS, cells expressing labeled mitochondria or MAVS were imaged and processed in FIJI by enhancing contrast and mean-filtering to generate an accurate mask of the location of mitochondria or MAVS within each cell. The mask was then dilated twice to the region immediately surrounding the mitochondria, and regions within the first dilation excluded from the mask, leaving only cytosolic regions near, but not adjacent to, the mitochondria or MAVS. The mean mNG signal within both masks was then measured and the ratio of the two values (Mito/Outer mito) plotted for each cell. These RIG-I-containing bodies are defined as being at least 1 μm in diameter and at least ten times brighter than the mean RIG-I signal throughout the cytosol.

#### SeV DI RNA Quantification

To better quantitate exactly how many RNA ligands are required to stimulate RIG-I signaling, we titrated cells with SeV and, following total RNA extraction, performed RT-qPCR for the SeV defective interfering (DI) RNA, which is the primary RIG-I ligand in SeV infection of A549 cells,^44–46^ and for IFN-β mRNA. To obtain an absolute SeV DI RNA copy number, we normalized to an *in vitro* transcribed (IVT) fragment of the DI RNA. In validation of our assay, we detected as few as 10^4^ copies of the IVT DI RNA, which corresponds to a limit of detection of ~0.5 DI RNAs per cell. All SeV-infected RNA extracts, but not mock-infected cells, had SeV DI RNA levels above this limit of detection. In brief, a 250 nucleotide SeV DI RNA standard was *in vitro* transcribed from DNA^47^ and purified by polyacrylamide gel. Purification of the full-length product was confirmed via BioAnalyzer pico gel (Agilent), and RNA concentration was determined via Qubit RNA Assay (Thermo Fisher). A549 cells were infected with indicated concentrations of SeV, and total RNA was extracted from SeV-infected cells using TRIzol. Following total RNA purification, SeV DI RNA was selectively reverse-transcribed with 50 nM gene-specific primer (AGTCCAAGACTATCTTTATCTATGTCCAC) from both RNA extracts and standard curve reverse-transcribed using MarathonRT. cDNA was purified qPCR was performed using LightCycler 480 Mastermix (Roche) using the following primers: SeV [Fwd: TCAGGTTCCTGATTTCACG, Rev: CCAAGACTATCTTTATCTATGTCCACAAG].

### QUANTIFICATION AND STATISTICAL ANALYSIS

Two-way comparisons were performed via student’s T test and comparisons between more than two conditions via one-way ANOVA in GraphPad Prism.

## Supplementary Material

1

## Figures and Tables

**Table:** KEY RESOURCES TABLE

REAGENT or RESOURCE	SOURCE	IDENTIFIER
Antibodies		
RIG-I (D14G6) Rabbit mAb	Cell Signaling Technology	3743; RRID AB_2269233
GAPDH Mouse mAb	SCBT	Sc-47724; RRID AB_627678
RIG-I Mouse mAb	Millipore Sigma	MABF297; RRID:AB_2650546
MAVS/VISA Rabbit mAb	Bethyl Laboratories	A300-782A; RRID:AB_669744
AlexaFluor555-conjugated Goat anti-Mouse Ab	Invitrogen	A32727; RRID:AB_2633276
AlexaFluor555-conjugated Goat anti-Rabbit Ab	Invitrogen	A32732; RRID:AB_2633281
AlexaFluor647-conjugated Goat anti-Rabbit	Invitrogen	A32733; RRID:AB_2633282
HRP-conjugated Goat anti-Mouse	ThermoFisher	G-21040; RRID:AB_2536527
HRP-conjugated Goat anti-Rabbit	ThermoFisher	31460; RRID:AB_228341
Bacterial and virus strains		
Sendai Virus (Cantell strain)	Charles River Labs	10100774
DH5-alpha E. Coli	New England Biolabs	C9287
Chemicals, peptides, and recombinant proteins		
Opti-MEM, Reduced Serum Medium	ThermoFisher	31985070
Ham’s F-12 Medium	ThermoFisher	11765047
Dulbecco’s modified eagle medium (DMEM)	ThermoFisher	11965092
Lipofectamine 2000	ThermoFisher	11668030
RNAiMAX	ThermoFisher	13778030
MitoTracker CMXRos	ThermoFisher	M7512
MitoTracker DeepRed	ThermoFisher	M22426
HaloTag OregonGreen Ligand	Promega	G2801
Interferon-alpha, recombinant	Millipore Sigma	SRP4596
SuperaseIN RNase inhibitor	ThermoFisher	AM2694
MarathonRT	Zhao et al.^39^	N/A
Gibson Assembly Mastermix	NEB	E2611
Alt-R S.p. Cas9 Nuclease V3	IDT	1081058
TRIzol	ThermoFisher	15596026
Lightcycler 480 SYBR green mastermix	Roche	04707516001
Superscript III	ThermoFisher	18080044
T7 RNA polymerase mutant P226L	Addgene	174866
Critical commercial assays		
Dual luciferase reporter assay system	Promega	E1910
Flp-In Core System	ThermoFisher	K601002
Experimental models: Cell lines		
A549	ATCC	CCL-185
A549, RIG-I k.o.	Invivogen	A549d-korigi
A549, mNG-RIG-I	This paper	N/A
A549, Halo-RIG-I	This paper	N/A
Huh7.5	Gift from Brett Lindenbach	N/A
HEK293T	ATCC	CRL-3216
FlpIn HeLa	Gift from Craig Crews	
Oligonucleotides		
ppp- and OH-SLR14: GGAUCGAUC GAUCG UUCG CGAUCGAUCGAUCC	Linehan et al.^8^	N/A
RIG-I sgRNA: TTGCAGGCTGCGTCGCTGCT	IDT	N/A
S. p Cas9 tracrRNA	IDT	N/A
Random hexamers	ThermoFisher	SO142
IFNB qPCR F: GCGACACTGTTCGTGTTGTC	ThermoFisher	N/A
IFNB qPCR R: GCCTCCCATTCAATTGCCAC	ThermoFisher	N/A
RIG-I qPCR F: CTGATTGCCACCTCAGTTGC	ThermoFisher	N/A
RIG-I qPCR R: GTCCCATGTCTGAAGGCGTA	ThermoFisher	N/A
ACTB qPCR F: TTC6CAGCAGATGTGGATCAG	ThermoFisher	N/A
ACTB qPCR R: GGTGTAACGCAACTAAGTCA	ThermoFisher	N/A
SeV DI RNA qPCR F: CCTCAGGTTCCTGATTTCACG	ThermoFisher	N/A
SEV DI RNA qPCR R: CCAAGACTATCTTTATCTATGTCCACAAG	ThermoFisher	N/A
SeV DI RNA RT primer: AGTCCAAGACTATCTTTATCTATGTCCAC	ThermoFisher	N/A
Recombinant DNA		
pUNO1-RIG-I and fluorescent-tagged variants	Invivogen, This paper	pUNO1-hrig-i
pcDNA5-mNG-RIG-I	This paper	N/A
pUNO1-IRF3 and fluorescent-tagged variants	Invivogen, this paper	pUNO1-hIRF3
pRL-TK plasmid	Promega	E2241
IFN-β/Firefly luciferase plasmid	Ren et al.^40^	N/A
mNG-N1	Allele Biotech	ABP-FP-MNEONSB
Software and algorithms		
ImageJ	Schneider et al.^41^	https://imagej.nih.gov/ij/
Zen Imaging Software	Zeiss	N/A
FindFoci Image Analysis	Herbert et al.^42^	N/A
GraphPad Prism 7	GraphPad	http://www.graphpad.com/scientificsoftware/prism

## Highlights

- RIG-I is activated rapidly by small quantities of viral RNA
- Signaling relies exclusively on the resident non-induced pool of RIG-I receptors
- RIG-I signaling occurs without mass aggregation at the mitochondrial membrane
- IFN-induced RIG-I forms signaling-unrelated cytosolic aggregates
