# Supplementary material for: A rapid RIG-I signaling relay mediates efficient antiviral response
Source: Mol Cell. Author manuscript; Available in PMC 2023 Jan 8. (PMC9825657; doi:10.1016/j.molcel.2022.11.018)
Supplement: 1 [file NIHMS1858447-supplement-1.pdf]

Molecular Cell, Volume *83*

Supplemental information

A rapid RIG-I signaling relay
mediates efficient antiviral response

Daniel T. Thoresen, Drew Galls, Benjamin Götte, Wenshuai Wang, and Anna M. Pyle

Supplemental Figure 1

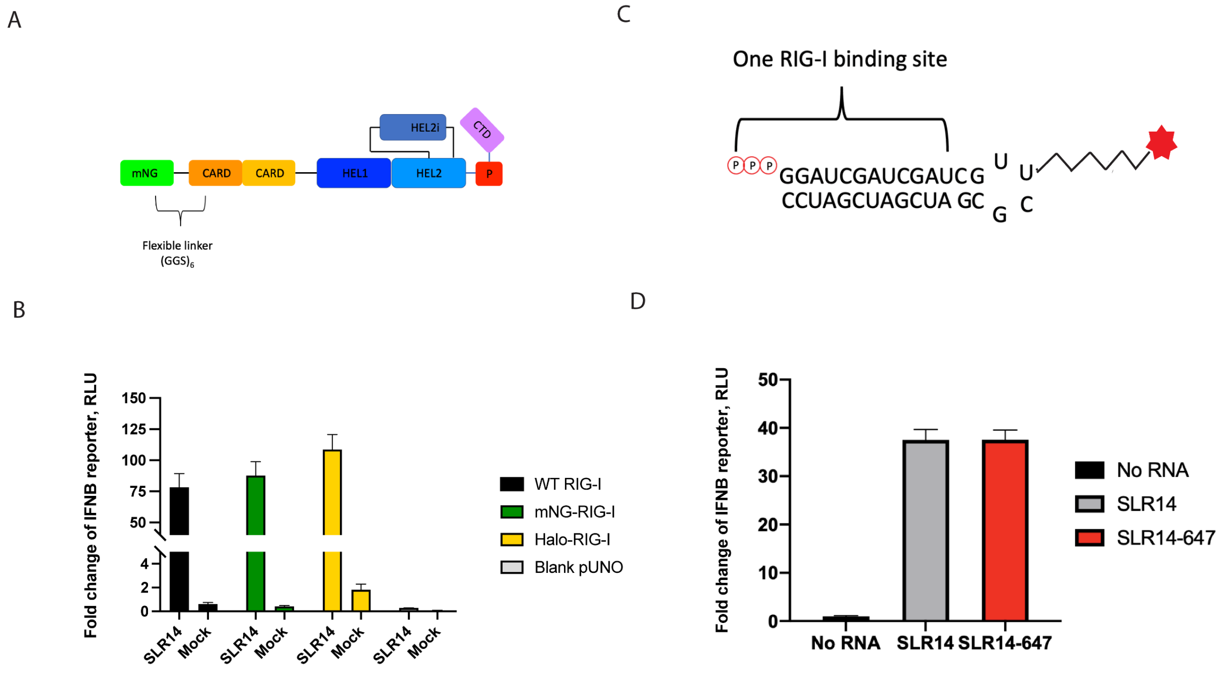

Supplemental Figure 2

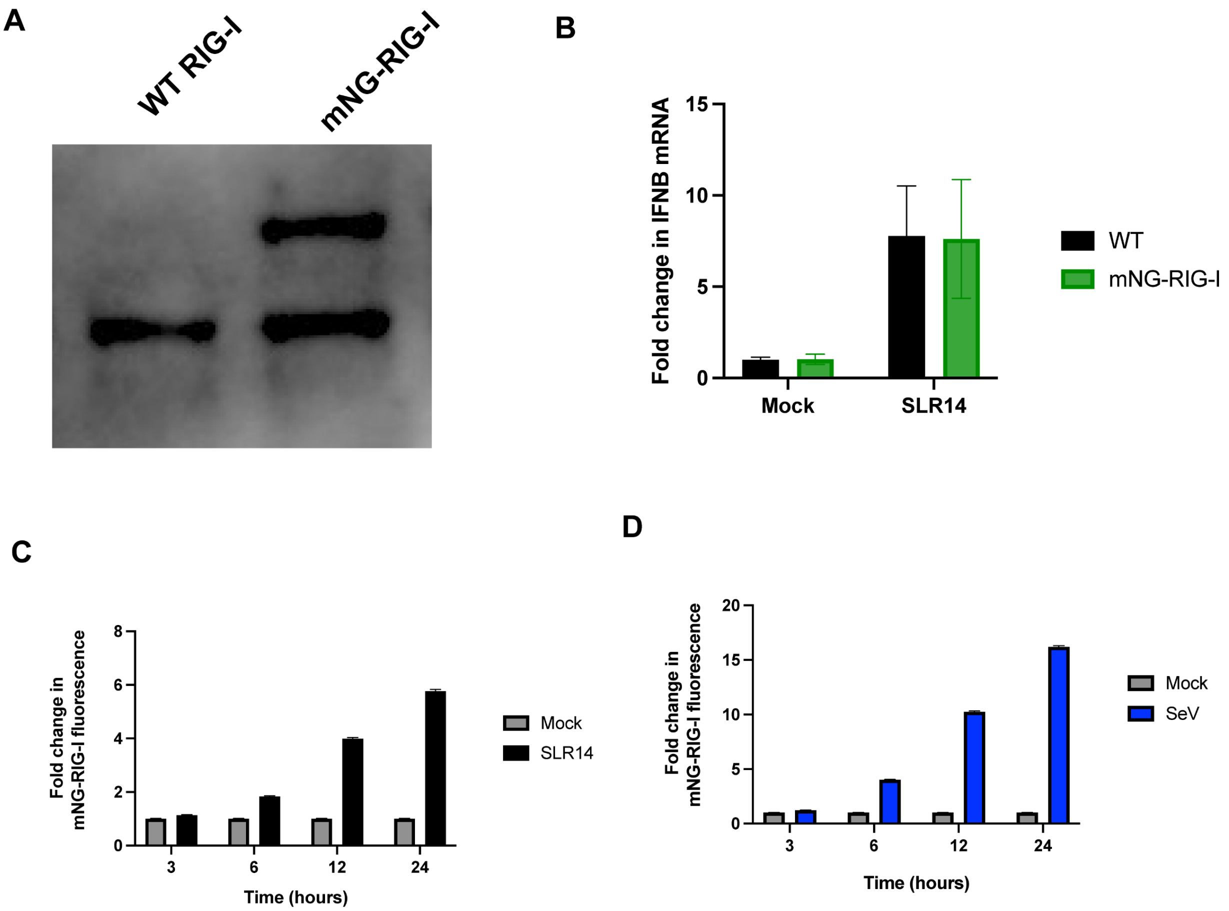

Supplemental Figure 3

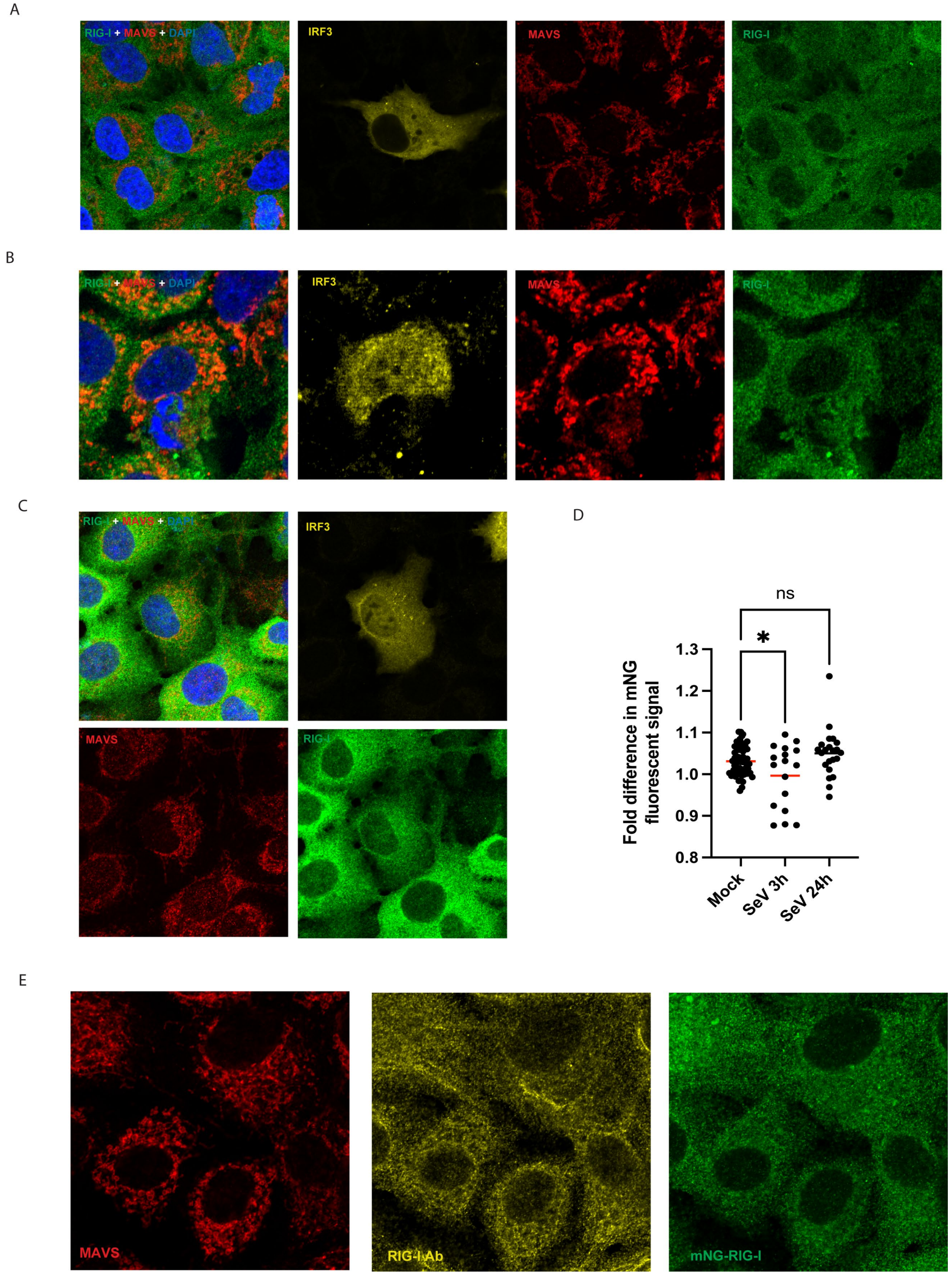

Supplementary Figure 1: Fluorescent labeling of both RIG-I and RNA has no effect on RIG-I signaling. Related to Figure 2.

(A) Schematic representation of the domains of RIG-I, and the location of the mNeonGreen fluorescent tag via N-terminal linker. **(B)** RIG-I signaling response in HEK293T cells expressing unlabeled RIG-I (WT RIG-I), mNG-RIG-I, Halo-RIG-I, or no additional RIG-I (Blank pUNO) was measured via relative luciferase activity at 12h following stimulation with 1 μg/mL SLR14 or mock transfection. **(C)** Schematic representation of the location of the fluorescent tag on SLR14. **(D)** RIG-I response in HEK293T cells expressing unlabeled pUNO-RIG-I was measured via relative luciferase activity at 12h following stimulation with 100 ng/mL SLR14 or SLR14-647. Data represent the means of three independent experiments. Error bars indicate standard deviation.

Supplementary Figure 2: Introduction of mNG tag to endogenous RIG-I in A549 cells does not disrupt signaling or expression. Related to Figure 3.

(A) Total cell lysate from unlabeled A549 cells (WT RIG-I) and mNG-RIG-I cells was collected and immunoblotted for RIG-I and GAPDH (not shown). **(B)** WT and mNG-RIG-I A549 cells were mock-transfected or stimulated with 100 ng/mL SLR14 for 6h prior to total RNA extraction, and the fold change in IFNB mRNA expression relative to mock-treated cells was measured via RT-qPCR. **(C and D)** mNG-RIG-I A549 cells were mock-treated or stimulated with 1 μg/mL SLR14 **(C)** or 100 HA units/mL SeV **(D)** for 3-24h prior to measurement of total cellular mNG fluorescence in 10,000 cells/condition via flow cytometry. The fold increase in mean cellular mNG fluorescence relative to mock-stimulated cells at 3h in each condition is shown. Data are means of three independent experiments. Error bars indicate standard deviation (B) or standard error of the mean (C).

Supplementary Figure 3: Immunolabeled RIG-I and MAVS do not display increased colocalization following RIG-I stimulation. Related to Figure 4.

(A) mNG-RIG-I A549 cells were transfected with pUNO-mCherry-IRF3 (yellow) 48h prior to fixation and immunolabeled with Rb anti-MAVS (red). **(B)** mNG-RIG-I A549 cells were transfected with pUNO-mCherry-IRF3 (yellow) 48h prior to fixation, infected with 100 HA units/mL SeV 3h prior to fixation and immunolabeled with Rb anti-MAVS (red). **(C)** mNG-RIG-I A549 cells were transfected with pUNO-mCherry-IRF3 (yellow) 48h prior to fixation, infected with 100 HA units/mL SeV 24h prior to fixation and immunolabeled with Rb anti-MAVS (red). **(D)** The mean RIG-I fluorescent signal directly overlapping MAVS and the mean RIG-I fluorescent signal 300 nm away from MAVS were calculated for each cell, and

the ratio of the two values was plotted for cells that were mock-infected (n = 60), SeV-infected for 3h (n = 17) or SeV-infected for 24h (n = 22). **(E)** mNG-RIG-I A549 cells were infected with 100 HA units/mL for 3h prior to fixation and immunolabeled with Rb anti-MAVS (red) and Ms anti-RIG-I (yellow).
